# Development of an attenuated vaccine against Koi Herpesvirus Disease (KHVD) suitable for oral administration and immersion

**DOI:** 10.1038/s41541-022-00525-6

**Published:** 2022-09-06

**Authors:** Sandro Klafack, Lars Schröder, Yeonhwa Jin, Matthias Lenk, Pei-Yu Lee, Walter Fuchs, Jean-Christophe Avarre, Sven M. Bergmann

**Affiliations:** 1 Friedrich-Loeffler-Institut, Federal Research Institute for Animal Health, Institute of Infectology, Greifswald, Germany; 2 Friedrich-Loeffler-Institut, Federal Research Institute for Animal Health, Institute of Molecular Virology and Cell Biology, Greifswald, Germany; 3 Friedrich-Loeffler-Institut, Federal Research Institute for Animal Health, Department of Experimental Animal Facilities and Biorisk Management (ATB), Greifswald, Germany; 4 GeneReach Biotechnology Corporation, Taichung, Taiwan; 5 ISEM, Univ Montpellier, CNRS, IRD, Montpellier, France

**Keywords:** Live attenuated vaccines, Herpes virus

## Abstract

Since the end of the1990ies, *Cyprinid herpesvirus 3* (also known as koi herpesvirus, KHV) has caused mass mortality events of koi and common carp all over the globe. This induced a high economic impact, since the KHV disease cannot be cured up to now, but only prevented by vaccination. Unfortunately, there is only one commercial vaccine available which is not approved in most countries. Therefore, there is an urgent need for new, safe and available vaccines. In this study, a live attenuated vaccine virus was generated by cell culture passages of virulent KHV, and shown to protect carp or koi after immersion or oral application against wild type challenge. An advantage of boost immunization was demonstrated, especially after oral application. Vaccination induced no or mild clinical signs and protecting antibodies have been measured. Additionally, the vaccine virus allowed differentiation of infected from vaccinated animals (DIVA) by PCR. The attenuation of the newly generated vaccine was tracked down to a partial deletion of open reading frame 150. This was confirmed by the generation of engineered ORF150 deletion mutants of wild-type KHV which exhibited a similar attenuation in vivo.

## Introduction

Common carp (*Cyprinus carpio* L.) is one of the most important freshwater fish in the world. In 2019 common carp was one of the most produced freshwater fish in the World, with 9.6% of the produced bio mass in aquaculture^1^. Carp production is an important economic factor, especially in Asia, where 4.15 million tons of common carp were produced, that equals 94.12% of global production^1^. Beside common carp, the ornamental variety from Japan, the koi, has a growing importance with enormous financial values for hobbyists. Since 2000 common carp and koi have been endangered by an *Alloherpesvirus*, genus *Cyprinivirus*, named *Cyprinid herpesvirus 3* (CyHV-3) or koi herpesvirus (KHV)^2,3^. The virus causes severe fatality rates of up to 100%^2^ in both varieties. Beside high mortality, lifelong persistence or latency and a wide host range for asymptomatic KHV infections are severe problems for carp production and koi breeding worldwide. Once the host is infected, KHV persists in it for the entire life span, like all other herpesviruses^4^. If carp become latently infected, the virus can be re-activated by stress, for example induced by netting as a part of normal procedures in fish handling. This can lead to virus re-activation and to a renewed shedding of infectious virus^5^. In Germany, it is common practice to transfer carp before winter in deeper ponds for wintering and in spring back to shallow ponds for growing and fattening. At the end, usually after three years of the production cycle, carp are captured by netting and kept in concrete ponds for fastening, until selling. These procedures can lead to a re-activation of a persistent infection resulting in tremendous losses in the carp population. Moreover, carp are usually held together with other species like tench (*Tinca tinca*), crucian carp (*Carassius carassius*) or pike (*Esox lucius*). Even though all these species can be infected by KHV without showing any clinical symptoms^6^, they can transmit infectious KHV to the only known susceptible species for koi herpesvirus disease (KHVD), common carp and the koi^2,6–8^.

Due to the high economic impact of KHV, efficacious and safe vaccines are mandatory and urgently needed to protect animal welfare and aquacultures. Up to now, there is no licenced commercial vaccine available in major parts of the world. However, one commercial vaccine was produced by the company KoVax in Israel, which was used for carp. This vaccine contains live attenuated virus, which was obtained after 26 serial cell culture passages, followed by UV treatment^9^. Unfortunately it seems to be virulent to small carp, weighing less than 50 g^10,11^. Additionally it cannot be excluded until today, that this vaccine might revert or recombine to a more virulent phenotype, because the precise reason for its attenuation is still unclear^10,12^.

Live attenuated vaccines seem to be the best choice for use in aquaculture of carp. The immune system is stimulated by the vaccine replication in the host, while inactivated vaccines lost this feature. As a consequence, cytotoxic T cells are not stimulated. Additional strong adjuvants and several applications of the vaccine are needed^12^. Until now, there are some vaccines developed but none of them is approved in EU or is commercially available, except the one from Israel. Most of these vaccines are live recombinant vaccines with a deletion of one or more genes of KHV genome^13–15^. One promising subunit vaccine was developed in Japan, by the use of liposomes as transport vehicles. KHV particles inactivated by formalin and incorporated into liposomes were used for vaccination, achieving 70% surviving carp after challenge^10^. Unfortunately, this vaccine is not available. More recently a Chinese group published their results on a combined DNA and protein subunit vaccine, based on ORF131 of KHV, achieving less than 80% protection^16^.

The biggest problem in designing vaccines against KHV is the missing knowledge on its major antigens and virulence factors. The proteome, the secretome^17,18^ and the immune response^18–24^ were investigated. However, the function of most of its 156 ORFs is still unclear. KHV possesses the biggest known herpesviral genome with 295 kbp and 156 predicted ORFs^25^. It has similarity to two other viruses of its genus, 36.6% similarity to CyHV-1 (carp pox virus) and 40.37% identity to CyHV-2 (goldfish herpesvirus).

In this study, a live attenuated KHV mutant was established by serial cell culture passage of virulent wild-type virus leading to a deletion of ORF150. Targeted deletion of this gene confirmed its relevance as virulence factor. Our results strongly indicate that ORF150-deleted KHV can be used as an efficacious and safe vaccine in carp production.

## Results

Before the present study was preceded, serial virus passages of a moderately virulent KHV isolate from Taiwan (KHV-T)^26^ were done in common carp brain (CCB) cells^27^ to further attenuate this virus. Usually attenuation by cell culture adaptation is done in cells from another than the natural host and target species of the desired vaccine. However, sufficiently efficient replication of KHV could be so far only achieved in cell cultures prepared from *Cyprinus carpio*, and is restricted to temperatures below 30 °C, usually between 20 °C and 26 °C. Next, it was necessary to test different CCB cell passages of KHV-T for virulence and their suitability as vaccine. In order to reduce the number of animal experiments, a recently published phylogenetic method^28^ was used to get a hint, which viruses might be suitable. Briefly, eight variable number tandem repeats (VNTRs) were used to estimate the phylogenetic distance between virus variants enriched in randomly selected passages. The virus exhibiting the most differences compared to the initially dominating virulent wild-type, might also be the most attenuated. Based on this, it was decided to use KHV-T from passage 51 and 78 because these passages appeared the most distant^28^ compared to their origin. Additionally, KHV from final passage 99 was used. This virus had the highest number of cell culture passages, so it should be well adapted to cell culture, and, thereby, might have lost its in vivo virulence.

### In vivo testing of different KHV-T passages

**Fig. 1:**
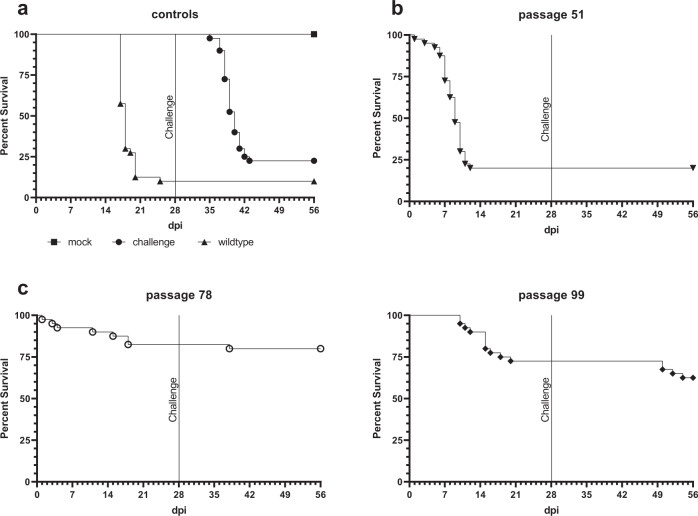
In vivo testing of different KHV-T passages. Survival data of carp infected with different CCB cell passages of KHV-T **b**–**d** compared to wild-type or mock infected animals **a**. The carp were infected with 10^5^ TCID_50_/mL of the indicated passage and, except one control group, challenged after 28 dpi with the same dose of wildtype KHV-T.

For in vivo experiments, six animal groups were established. Each group consisted of 40 randomly selected juvenile carp (age between six and nine month) and five one-year-old carp. Three groups were infected/immunized by immersion with 10^5^ TCID_50_/mL of KHV-T passage 51, 78 or 99, respectively. One positive control group, infected with wild-type KHV-T, and one negative control group, without any treatment, was included. Additionally, a second group without any initial treatment was set up to verify the success of challenge infection. As expected, no mortality or morbidity was found in the untreated control (Fig. 1a, rectangles), whereas between day 7 and 25 after KHV-T wild-type infection, 36 juvenile carp died from the positive control group, representing a 90% mortality rate (Fig. 1a, triangles). Moreover, a high morbidity was observed. This was indicated by clinical signs like increased mucus production or total loss of mucus (sandpaper skin) and haemorrhages and white patches on the skin. Comparable results were achieved in the challenge control group, where 77.5% of the infected fish died between day 39 and 48 of the experiment, corresponding to 7^th^ and 14^th^ day after infection (Fig. 1a, circles).

The first tested virus was KHV from passage 51 (KHV-T P51). Here, only 9 juvenile carp survived until day 12 post infection (dpi), resulting in a mortality rate of 77.5% (Fig. 1c). All fish expressed typical symptoms, like the positive control fish. However, no more clinical signs were observed at 15 dpi, short after mortality had stopped. The surviving carp also survived challenge without any clinical signs.

KHV from passage 99 (KHV-T P99) was significantly less virulent than the wild type virus. It led to a mortality of 27.5% between 9 and 20 dpi. (Fig. 1d). No additional fatalities were observed until day 21 post challenge, but after this day four additional carp died, leading to a total mortality of 37.5% (15 of 40 carp). Moreover, 100% of the carp expressed symptoms (increased mucus, skin lesions and haemorrhages) beginning from 10 dpi. However, six days after symptoms manifested, they disappeared. After wild type challenge, moderate clinical signs were observed twice in 4 (33 dpi) or 2 (43 dpi) affected carp.

In contrast, the mortality induced by passage 78 (KHV-T P78) was only 17.5% until 28 dpi (Fig. 1b), which was a reduction of 72.5% compared to wild type infection. Amazingly, all carp were always without any clinical signs, confirming the attenuation of the tested virus. After wild type KHV-T challenge, only one additional fish died at 37 dpi. Thus, the total mortality was 20% (8 out of 40 carp). In four of the carp vaccinated with passage 78, moderate symptoms like increased mucus and weak haemorrhages appeared between 4 and 11 days after challenge (32 to 39 dpi), resulting in a morbidity of 10%.

### Improvement of vaccination procedure with KHV-T P78

**Fig. 2:**
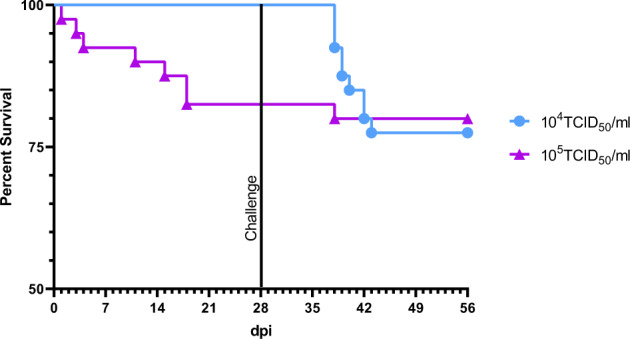
Improvement of vaccination procedure with KHV-T P78. Dose dependence of virulence and protective efficacy of KHV-T P78. Carp were infected by immersion with 10^4^ TCID_50_/mL of KHV-T P78, and challenged with 10^5^ TCID_50_/ml of wild type KHV after 28 days. Survival rates in this experiment (blue line), and in the previous study with 10^5^ TCID_50_/ml of KHV-T P78 (purple line) are compared.

Since KHV-T P78 was apparently the most promising vaccine candidate in the initial studies, we focused our further work on it. In the next experiment, immunization with a tenfold reduced concentration (10^4^ TCID_50_/mL) of the selected virus was tested. After infection with the lower dose of KHV-T P78, no mortality and no clinical signs were observed (Fig. 2). However, after wild type KHV-T challenge, a mortality rate of 30% between days 33 to 46 post vaccination was recorded, which was even higher than the cumulative mortality in the previous vaccination experiments with 10^5^ TCID_50_/mL of KHV-T P78.

**Fig. 3:**
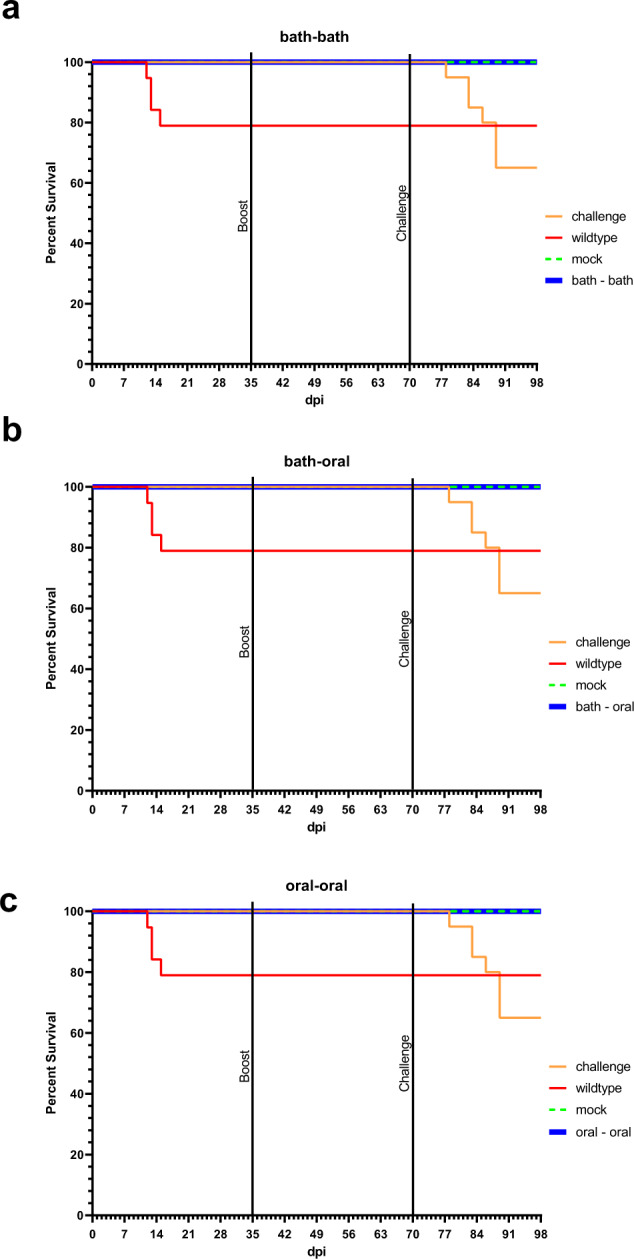
Comparison of vaccine application procedures. Carp were prime immunized by immersion in 10^4^ TCID_50_/mL of KHV-T P78 **a**, **b** or by feeding for 3 days with a total of 10^9^ TCID_50_ of KHV-T P78 encapsulated in alginate **c**. Five weeks later boost immunization was done with the same procedure, by immersion **a** and orally **b,** **c**. Challenge infection was performed by immersion in 10^5^ TCID_50_/mL with wild type KHV-T at 70 dpi. Immunized fish (blue line) survived challenge infection without any documented mortality.

Thus, further improvement was necessary. Since prime-boost vaccination is a common practice, we decided to apply KHV P78 twice to improve protection. For this purpose, carp were immunized by immersion in water containing 10^4^ TCID_50_/mL for 1 h. After 35 days this procedure was repeated. At day 70 post immunization, carp were challenged with wild type KHV. All prime and boost vaccinated carp survived immunization as well as challenge infection, whereas more than 20% of the control animals died. After prime immunization, 50% of carp expressed clinical signs like increased mucus or haemorrhages, which disappeared after one week. Following the boost immunization, no morbidity was observed. Even after challenge infection, only three carp showed very weak clinical signs of KHVD (Fig. 3a).

In the next step, we tried to use oral immunization as a boost technique. In this case, the carp were fed once a day for three days (33–35 dpi) with the vaccine candidate. The virus was encapsulated into alginate for this purpose. At each of the three days 3.3 × 10^8^ TCID_50_, in total 10^9^ TCID_50_ were given to each treated group, which is an equivalent of 10^5^ TCID_50_/mL in 10 L immersion. Again, the carp were challenged at 70 dpi. All carp survived the procedure. This time only 10% of the fish had mild signs of a KHVD, after prime immunization (Fig. 3b). Lastly, we tried to immunize the fish exclusively by oral application. Again, the treatment was done over three days with 3.3 × 10^8^ TCID_50_ on days 0–2, and 33–35, as described before. After 70 days carp were challenged with wild type KHV-T. All carp survived this trial without any clinical signs after vaccination while two carp had mild clinical signs after challenge (Fig. 3c).

**Fig. 4:**
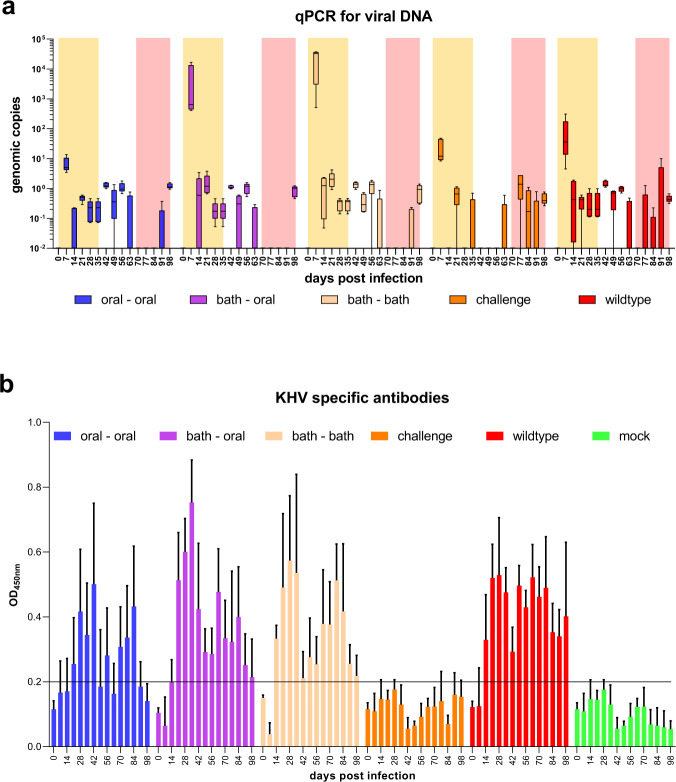
Genetic and serological data of different vaccine applications. Viral load in gill swabs was recorded over the whole time of the trial by qPCR detection of viral DNA **a**. The first five weeks (prime) are marked by a yellow background, while a white background is used for the next five weeks (boost), and the red background for the last five weeks after challenge. Boxplot: center line—median, limits—25th and 75th percentiles, whiskers—minimum and maximum. Humoral immune response was determined by ELISA **b**. The assay on plates coated with virus particles detects KHV-specific antibodies, which are present after vaccination/infection. Error bars represent standard deviation.

Additional to morbidity and mortality blood samples and gill swabs were analysed (Fig. 4). With these samples the humoral immune response and viral loads were quantified. At all time points after immunization / infection, viral DNA was detected by qPCR (Fig. 4a). Furthermore, as expected, all mock infected carp had no KHV-specific antibodies during the experiment. The highest viral loads were observed 7 d after wild-type infection of naive carp, and after the first immunization with KHV-T P78 by immersion. In contrast, only low amounts of virus were shed after oral vaccination, which correlated with the absence of clinical signs. KHV-specific antibodies were found in all wild-type infected or vaccinated groups from two weeks after primary infection until the end of the experiment. In the vaccinated groups KHV-specific antibody titres increased until four to five weeks after prime immunisation, decreased until seven weeks p.i., and then re-increased as a consequence of boost vaccination (Fig. 4b, Table 1).

**Table 1:** Oligos used for real-time-RT-PCR of KHV infected CCB cells.

Primer	Sequence (5’ -> 3’)	source
IL-1b_for	GTAACGTGTGCCGGTTTCTT	^65^
IL-1b_rev	GCAACACAAAAGGAAGCACA	
TNFa_for	GCTTGTAGCTGCCGTAGGAC	^65^
TNFa_rev	GGTGGCTTGGAATTAGTG	
NF-kB_for	TGGCTGGAGAGGATCCATAC	^65^
NF-kB_rev	AAAGCCCCTCTGTTTTGGTTG	
40 S ribo. S11_for	CCGTGGGTGACATCGTTACA	^20^
40 S ribo. S11_rev	TCAGGACATTGAACCTCACTGTCT	
Beta-actin_for	TCACACCACAGCCGAGAG	^20^
Beta-actin_rev	CAGGGAGGAGGAGGAAGCAG	
IL-6_for	TGAAGACAGTGATGGAGCAGCAGA	^66^
IL-6_rev	CCTCACAGCAATGTGGCGAACA	
IL-10_for	TGATGACATGGAACCATTACTGG	^67^
IL-10_rev	CACCTTTTTCCTTCATCTTTTCA	

### Impact of ORF150 on Virulence

**Fig. 5:**
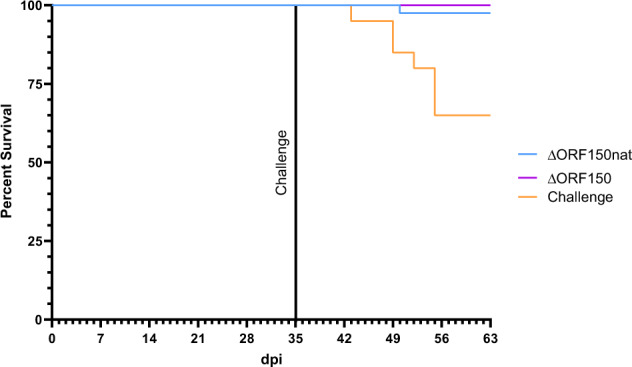
Impact of ORF150 in vivo. Direct impact of ORF150 deletion on virulence of KHV. Carp were infected by immersion with 10^5^ TCID_50_/mL of engineered KHV-T recombinants possessing a complete deletion of ORF150 (purple line), the naturally occurred partial deletion of ORF150 inserted into wildtype background (blue line), or challenge infection wildtype KHV-T (orange line). Shown are the survival rates after wild-type challenge at day 35 p.i.

Since KHV-T P78 induces protective immunity in carp, the genetic background of this virus had to be analysed. As published previously^29^, a virus mutant possessing a partial deletion of *ORF150* was highly enriched in this passage, and we assumed that this deletion might have been responsible for the attenuation of KHV. Therefore, KHV-T recombinants possessing either the naturally occurred deletion (Δ*ORF150*nat), or a complete deletion of *ORF150* (Δ*ORF150*) were prepared from the unpassaged wild-type virus. Two groups of carp were infected by immersion with 10^5^ TCID_50_/mL of the respective virus and challenged with wild type virus at 35 dpi (Fig. 5). During this trial, 40% of KHV Δ*ORF150*nat – treated carp developed symptoms, whereas only 10% of KHV Δ*ORF150*- treated carp had symptoms. These clinical signs were mainly characterized by increased mucus and disappeared until day 42. Moreover, all carp survived vaccination and challenge. Only one carp of KHV Δ*ORF150* group died 49 d p.i., most likely as a consequence of sampling one day before. In contrast, more than 25% of a naive control group succumbed to challenges.

### Influence of ORF150 deletion on host immune response

**Fig. 6:**
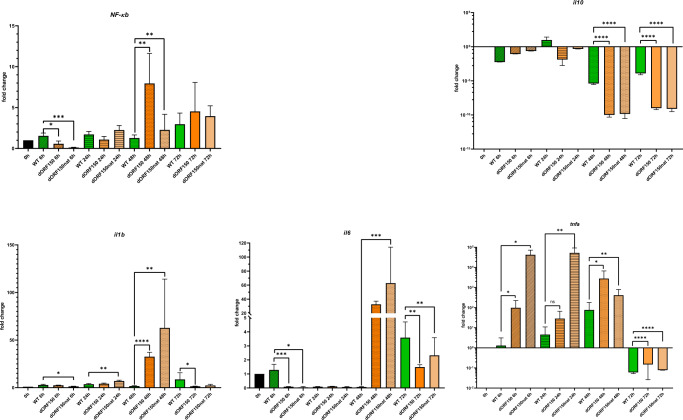
Expression profile of certain host genes. mRNA expression profile of affected genes in CCB cells after KHV infection. Green bars represent wildtype KHV infection, orange bars represent ΔORF150 KHV infection and pale orange bars represent ΔORF150nat KHV infection. All samples were prepared as triplicates. Expression profile was measured by q-RT-PCR using SYBR Green. Data are normalized to actb and rps11 expression levels. Error bars represent standard deviation. ns: not significant, *p ≤ 0.05, **p ≤ 0.01, ***p ≤ 0.001, ****p ≤ 0.0001, significance test: students t-test.

In addition to viral attenuation by ORF150 deletion we tested the impact of *ORF150* on the immune response. Considering that pORF150 might be an Ubiquitin E3 ligase^29^, we assumed an effect on cellular signalling, which depends on ubiquitination. Therefore, we tested several regulatory host genes for differential expression after infection of CCB cells with wild-type or ORF150-deleted KHV at a multiplicity of 5, and identified *NF-κB* as a possible target of pORF150. After 48 h infection of CCB cells with both KHV mutant’s gene expression of *NF-κB* was significantly (*ΔORF150* 7-fold, and *ΔORF150*nat 2-fold) increased compared to wild-type infected cells (Fig. 6). NF-*κB* regulated genes were also tested. Here we found that the expression of proinflammatory cytokines *IL-1β* and *IL-6* was increased 48 h after infection, but not at later times. In contrast, the expression of anti-inflammatory *IL-10* was decreased after 48 and 72 h in the absence of the ORF150 gene product. In particular, the expression of *TNF-α* was substantially increased in cells infected with the ORF150 mutants.

## Discussion

KHV poses a massive and serious threat for carp and koi production worldwide^30^. Even if the fish survives the disease, it will carry the virus life-long, and will transmit the virus to other susceptible animals, which may cause outbreaks of KHVD. Thus, it is important to generate an efficient vaccine, which leads to solid protection against disease, even if it cannot completely prevent superinfection of immunized fish by wild type KHV in the environment. Keeping this in mind, it is obvious that the virus originated from passage 51 of KHV-T was not suitable as vaccine, since it is a potent killer with wildtype virus characteristics. It induced mortality in the groups of infected fish even faster than the wild type virus. In less than 14 dpi, more than 75% of carp in this group died. Particularly, the early acute symptoms with high impact, e.g. severe skin bleedings, loss of mucus and skin lesions, indicated a short incubation time inducing a peracute disease, comparable to wild-type virus infection. In contrast, passage 99 presented first features indicating attenuation. Compared to wild-type KHV, the mortality was drastically reduced to only 25% losses after primary infection, combined with a short-peaked morbidity around 10 dpi. However, a drawback of this vaccine candidate was the incomplete protection, leading to additional fatalities after challenge the fish with wild-type KHV-T.

Compared to the other viruses, KHV-T P78 was the most promising vaccine candidate tested in this experiment for virulence and immunogenicity. Infectious doses of 10^4^ TCID_50_/mL and 10^5^ TCID_50_/mL caused fewer damage, than the other tested viruses. A protective immunity was achieved, when carp were immunized with 10^5^ TCID_50_/mL of KHV-T P78. However, several carp got sick and died after application of this virus dose. In contrast, with 10^4^ TCID_50_/mL of same virus, no fish died until challenge, albeit few fatalities were observable after challenge. As a result, this virus might be useful as a vaccine, if the immunization dose is low enough to cause no mortality, like 10^4^ TCID_50_/mL, and the immunity is safe enough, as induced with 10^5^ TCID_50_/mL. This could be achieved by prime/boost immunization. In this modified protocol both immunizations were performed as before, by immersion in 10^4^ TCID_50_/mL KHV-T P78. This time, all fish survived immunization, and more importantly, they survived challenge infection without losses, indicating an improved immunity. In the following step, we tried to further improve our boost vaccination approach by oral administration. To this end, the virus suspension was encapsulated into alginate and fed to the fish for three consecutive days. Again, all fish survived. Finally, we tried to perform both immunization step orally. Once more all fish survived this procedure. Moreover, at no time after this kind of immunization the carp expressed any clinical signs of KHVD. This implies a better tolerability of the oral vaccination compared to the immersion. Furthermore, oral vaccination is easier to apply in aquaculture facilities, since no direct interaction with the fish, like catching and touching, is necessary^31^. This improves animal welfare, and reduces the risk of spreading the pathogens. Fish can be vaccinated by administration of oral vaccines in spring, before water temperature is rising and KHV is spreading in aquaculture. However, for commercial production of KHV-T P78 a cloned variant will improve safety a reduces the risk of reversion. Despite of many previous attempts to produce a protective vaccine, none of them resulted in a generally accepted, commercially available one up to now. Recently, a subunit vaccine, based on pORF131, was tested with prime-boost regime and oral application^16^. However, the protective efficacy was not more than 67%. Another approach used a vaccine based on ORF25 for DNA vaccination^32^. Here protective immunity was achieved by three subsequent injections of the respective plasmid, and observed after challenge by immersion. However, if the carp were challenged by cohabitation, no protection was achieved. Furthermore, the DNA vaccine turned out to be not suitable for oral vaccination.

One of the first specific immune reactions against a pathogen is the generation of antibodies. In fish sera, the main antibody type is tetrameric IgM^33^. Neutralisation is a very important function of antibodies, which is achieved by blocking of viral receptor-binding proteins to prevent attachment to the host cell surface. Opsonisation is another important function of antibodies which results in tagging infected cells for the innate immune response^34,35^, e.g. for phagocytes. In the trials described here, we detected KHV-specific antibodies 14 to 21 days after prime immunization (Fig. 4b), which was in line with previous studies^14^. This was used for the second immunization after five weeks to boost specific antibody response. As expected, the boost vaccinations after five weeks consumed first the available antibodies, documented by decreased antibody titres. However, in the following weeks, the titres increased again and contributed to protective immunity.

From previously published data^29^, we knew that KHV-T P78 possesses a deletion in ORF150, which might completely abolish its expression, or lead to a truncated, presumably non-functional protein. As mentioned before ORF150 might code for an ubiquitin E3 ligase^29^, which is the last enzyme responsible for ubiquitination^36^. Ubiquitin is a small protein, which can be added to proteins^37^ and has an influence on protein stability^38,39^, on the tolerance towards DNA damage, inflammatory immunity, endocytosis and ribosomal protein synthesis^40^. Moreover, it was shown, that ubiquitination is involved in the immune evasion or establishment of a viral infection. In case of HIV, the viral protein Vif influences ubiquitination, which leads to degradation on Vif and the anti-viral protein APOBEC3G^41^. ICP0, an E3 ligase of HSV-1, prevents the activation of TNF-α^42^. It is known that herpesviruses established many ways to work against the host innate immune response^43^. As published earlier, KHV downregulates the interferon (IFN) response of carp^20^. IFNs are known to work against viral infection, e.g. by inhibition of viral mRNA synthesis^44,45^. As previously mentioned, pORF150 might interfere with the anti-viral response of the carp. Using KHV recombinants completely lacking *ORF150* (ΔORF150) or possessing the naturally occurred of truncated gene of KHV-T P78 (ΔORF150nat) we have now shown that these mutations lead to a different gene expression of at least NF-*κB*, *il6*, *il10*, *il1b, and TNF-α than* after wild-type KHV infection (Fig. 6). The NF-*κB* pathway is a well-known for its regulation by ubiquitination^46–48^, and, notably, many viruses employ mechanisms for interfering with this pathway^42,49^. Using the generated virus recombinants, we also wanted to verify the importance of *ORF150* for KHV virulence. Both virus recombinants proved to be highly attenuated in animal trials, and a single immunization with them was sufficient to induce a protective immunity in carp against wild type challenge (Fig. 6). Only one loss was recorded in the group treated with KHV-T ΔORF150. However, this loss was not clearly related to the challenge infection, because the fish died one day after sampling without any clinical signs Thus, the in vivo trial with the engineered ORF*150* deletion mutants confirmed the importance of the gene or its products for the virulence of KHV and the suitability of such mutants as live vaccines. First in vitro studies were performed to elucidate the biological function of pORF150. CCB were infected with KHV-T ΔORF150, ΔORF150nat or wild-type virus and gene expression was investigated with a focus on NF-*κB* pathway and innate immune response. As mentioned before, herpesviruses evolved many ways to interfere with innate immunity by E3 ligases. Although replication kinetics and virus spread and of the two deletion mutants on CCB cells was undistinguishable from wild-type KHV-T (results not shown), differential expression was documented for *nfκb*, *il6*, *il10* and *il1b* 48 h after infection. Strikingly, proinflammatory genes, e.g. *nfκb*^50^, *il6* and *il1b*^51^, were increased, while anti-inflammatory *il10*^52^ was downregulated, when *ORF150* was missing or truncated. The highest increase in gene expression was documented for *tnfa*, which results in the proinflammatory cytokine TNF-α^53^. This leads to our assumption, that pORF150 inhibits the inflammatory response of carp to facilitate viral proliferation. Thus, deletion of ORF150 may result in less efficient in vivo replication of KHV which is responsible for attenuation. Furthermore, the ORF150 mutants and KHV-T P78 support proinflammatory immune response, which improves their efficacy as vaccines.

Earlier vaccination studies presented promising data. One of these studies was based on a purified and formalin inactivated KHV^10^. After purification, KHV was fused with liposomes, and carp were vaccinated orally. Although this inactivated vaccine was very safe, and first results were promising, no commercial vaccine was established. In contrast to live attenuated viruses, inactivated vaccines are not replicated in the host and cannot undergo mutation and reversion. For certain live vaccines, especially KV3 (KoVax, Israel), it has been shown that reversion to virulence is possible and dangerous^54^. Since mutation cannot be excluded in live vaccines, careful control of every vaccine batch is necessary, as well as investigation on genetic stability of the vaccine. Future studies are necessary to determine the biosafety of live attenuated KHV vaccines. Due to latency it cannot excluded that the attenuated variant will persist in aquaculture and environment, especially in non-target hosts e.g. pike, tench or crucian carp. The same will be done for the vaccine virus presented in this work, although the deletion of ORF150 excludes a simple reversion of the defective gene. In case of genetically engineered vaccines it might be possible to generate non-persistent vector vaccine, which are no longer limited by latency. Moreover, pond site trials are needed to approve the effectiveness of the presented viruses under real-life conditions. In summary, we could show, that spontaneous or engineered deletion of *ORF150* has a major impact on attenuation of KHV, and enhances the innate immune response. Finally, we demonstrated the use of alginate-encapsulated live attenuated virus as vaccine and the advantage of a boost immunization to achieve a robust and reliable immunity.

## Material and methods

### Cell cultivation and virus replication

Common carp brain cells (CCB, CCLV-RIE 0816)^27^ were propagated at 26 °C in minimal essential medium with Earls’ salts (Invitrogen) supplemented with 10% FBS, 10 mM HEPES, 2.2 g/L NaHCO_3_ (Roth), 1% non-essential amino acids (Biochrom) and 0.12 g/L pyruvic acid sodium salt (Merck). KHV isolates were added to 24-h-old CCB cell monolayers in the necessary volume of cell culture medium, and incubation at 26 °C was continued until a pronounced cytopathic effect (CPE) developed.

### Determination of virus titres

The titres were determined by the TCID_50_ assay. CCB cells were seeded into 96-well plates (Corning) and were incubated for 24 h at 26 °C with 2.5% CO_2_. Cell culture medium was removed and cells were covered with ten-fold serial virus dilutions in cell culture medium. After 7 days of incubation, the virus titres were determined by counting infected wells. The TCID_50_/mL values were calculated using the method of Spearman and Kaerber^55,56^.

### Initial vaccination experiments

For vaccination, groups consisting of 40 six-month-old carp and five one-year old carp per aquarium were used, according to European Pharmacopoeia monographs 1521, 1580, and 1581. All carp were kept at 20 °C in a re-circulating system with a daily water exchange of 10% of the entire volume. Health checks of fish were done daily. After transferring the fish into the aquaria, they were adapted for 14 days to the conditions. Initial sampling was done after adaption. Gill swabs were taken from 5 small carp, and gill swabs as well as blood were taken from five big carp per aquarium, sampling was always done non-lethal. Gill swab samples were taken from the smaller carp randomly. 24 h before sampling, fish were stressed by netting. Vaccination was performed by placing the fish in a 20 L vessel, containing 10 L of water, with 10^5^ TCID_50_/mL of one vaccine candidate (KHV-T P51 or P78 or P99). Oxygen supply was included. After one hour of vaccination by immersion, all fish were taken back by netting in their respective aquarium. The same procedure was done for KHV-T wild type virus infection which was used as a positive non-vaccinated control. Gill swabs and blood were sampled every two weeks of big five carp per group. On day 28 post vaccination all carp were challenged with 10^5^ TCID_50_/mL of wild type KHV-T. Additionally, an untreated group was also challenged with 10^5^ TCID_50_/mL of wild type KHV-T, which served as challenge control. After challenging, carp were kept for additional 28 days. Samples were taken again every two weeks. At 56 dpi/dpv, final samples were taken and experiment was terminated.

### Dosage effect of KHV-T P78

Animal experiments were conducted the same way as mentioned before for initial vaccination experiments. The only difference was the use of only KHV-T P78 at 10^4^ TCID_50_/mL (10^8^ TCID_50_ in 10 L).

### Prime-boost vaccination

Experimental set-up was similar to the initial experiments, with the exception that the control groups were reduced to 20 animals. Moreover, first immunization (prime) was performed with KHV-T P78 at a concentration of 10^4^ TCID_50_/mL (10^8^ TCID_50_ in 10 L) by immersion for one hour or by oral application of a total of 10^9^ TCID_50_, encapsulated into alginate, and given over three days instead of regular food, direct into the aquarium. Second immunization (boost) was performed after 35 days, in the same manner as prime immunization. Before feeding alginate capsules, fish were kept without additional food supply for 12 h.

### Encapsulation of KHV-T into alginate

Two grams of sodium alginate (Carl Roth, Germany) were dissolved in 100 mL of demineralized water. This alginate solution was mixed with an equal volume of KHV-T P78 suspension, to achieve a final concentration of one percent sodium alginate. Additionally, the colour of the capsules was adjusted to the colour of the regular food with food colouring. Finally, the alginate-KHV-solution was dropped into 1 M CaCl_2_-solution (Carl Roth, Germany), with a mean drop volume of 50 µL. The resulting capsules were rinsed once with isotonic sodium chloride and used for vaccination trials.

### Anti-KHV antibody ELISA

Serum was prepared by taking blood from the caudal vein, which coagulated in Microtainers (Becton Dickinson) overnight or at least for four hours. Serum separation was done following manufacturer’s instructions. Readily prepared serum was stored at −20 °C until use. Antigen was purified and the ELISA was proceeded as reported earlier^57^. Briefly, ELISA plates (Medisorp, Nunc) were coated with 0.3 µg of purified KHV per well in sodium bicarbonate buffer (3.5 g/L, pH 8.6) overnight. Afterwards, the plates were blocked with Roti®-Block (Carl Roth) and incubated at room temperature for 1 h. Plates were washed three times with PBS-T (0.05% Tween 20), remaining fluid was removed carefully. Carp sera were diluted in PBS-T and 100 µl/well were incubated on the plates for 1 h at room temperature. Then, plates were washed again three times with PBS-T and covered with 100 µL mouse anti-carp-IgM per well, diluted 1:128 (Aquatic Diagnostic) in PBS-T. After incubating for 1 h at room temperature, the plates were washed and covered with 100 µL 1:50,000 diluted anti-mouse-HRP conjugate (Sigma-Aldrich) per well and incubated for 1 h again. Three times washing was done before 100 µL per well of the substrate TMB/E (Merck) were added. The reaction was performed at room temperature and was stopped after 10 min with 100 µL per well 1 mol/L sulfuric acid. Then, the optical density was measured at 450 nm with iMark Microplate Absorbance Reader (Bio-Rad), values above 0.2 were regarded as positive.

### DNA extraction

Gill swabs were incubated in 500 µl Buffer K (100 mM NaCl, 10 mM Tris-HCl, 50 mM EDTA, 0.2% SDS, pH adjusted to 8.0) overnight at 56 °C with 10 U proteinase K (NEB). Then, 200 µL 5 M guanidinium thiocyanate were added and centrifuged for 1 min at 12,000 × *g*. Supernatant was mixed with 200 µL isopropanol and applied to silica columns (Epoche Life Science). After centrifuging for an additional minute at 12,000 × *g*, the spin column was washed with 75% ethanol in TE (10 mM Tris, 1 mM EDTA, pH 8.0). The dried column was placed in a new collection tube (2.0 mL, Eppendorf) and 50 µL of DEPC-treated water was added and incubated for 15 minutes. The spin column was finally centrifuged and the eluted DNA was stored at −20 °C until use.

### Detection of KHV–DNA by KHV qPCR

KHV DNA was detected as described previously^58,59^. Briefly, 12 µL reaction mix was prepared using GoTaq qPCR Master Mix (Promega). The mix included 2 µL of prepared DNA. For quantitation we used standard dilutions of a plasmid bearing a short KHV sequence^59^. All reactions were run at CFX96 Real-Time PCR Detection System (Bio-Rad).

### Construction of recombination plasmids

Genomic KHV-T DNA was prepared from infected CCB cell lysates as described^60^. The ORF150 gene region including nucleotides (nt) 256,992 to 260,908 of the KHV-T genome sequence (GenBank accession number # MG925491)^26^ was amplified by PCR using KOD Xtreme Hot Start DNA Polymerase (Merck), and primers KTO150R-HFF (5′-*AACGACGGCCAGTGAATTC*AAGATGTTGGCTGCGATG-3′) and KTO150R-HFR (5′-*CCATGATTACGCCAAGCTT*CGGCAAACTCTTCCCAG-3′). The 5′-terminal extensions of the primers (printed in Italics), overlapped with the ends of the *Eco*RI/*Hind*III-digested (restriction sites underlined) and Klenow-treated plasmid vector pUC19 (GenBank # M77789) which permitted ligase-free Hot Fusion cloning^61^.

For deletion of ORF150 codons 24 to 583 (of totally 630) the obtained plasmid pUC-KTO150R was doubly digested with *Sph*I and *Xho*I, treated with Klenow polymerase, and religated, resulting in pUC-KTΔO150. To facilitate identification of KHV recombinants, the reporter gene plasmid pUC-KTΔO150GFP was prepared by ligation of *Sph*I/*Xho*I-digested and alkaline phosphatase-treated pUC-KTO150R with an isolated 1636 bp *BamH*I/*Xho*I-fragment of pBl-GFP^62^ containing an expression cassette for enhanced green fluorescent protein (eGFP). After ligation of the *Xho*I fragment ends, the non-compatible overhangs created by *BamH*I and *Sph*I were blunted by Klenow-treatment, and ligation was continued.

Furthermore, a recombination plasmid containing the partial *ORF150* deletion, which naturally occurred during serial in vitro passage of KHV-T^29^ was prepared by using genomic DNA of P78 and primers KTO150R-HFF and -HFR for PCR amplification and subsequent Hot Fusion cloning. The insert fragments of the resulting plasmid pUC-KTΔO150nat, and of the other constructs were characterized by DNA sequencing.

### Generation of KHV recombinants

Virus mutants were obtained by homologous recombination between genomic and plasmid DNA, essentially as described before^14^. In the first experiment, CCB cells were co-transfected (K2 Transfection System, Biontex) with genomic KHV-T DNA and the reporter gene plasmid pUC-KTΔO150GFP. The co-transfection progeny was analyzed by plaque assays on CCB cells, and green fluorescent cell foci were picked by aspiration. After three successive plaque tests, the desired eGFP-expressing gene deletion mutant KHV-TΔO150GFP could be purified to homogeneity. Subsequently, genomic DNA of this virus recombinant was prepared and used for co-transfections with either pUC-KTΔO150 or pUC-KTΔO150nat. This time, the virus progenies were screened for reporter gene deletion mutants, and non-fluorescent virus plaques were aspirated. By repeated plaque assays, the deletion mutants KHV-TΔO150 and KHV-TΔ150nat were purified to homogeneity. Genomic DNA of all generated KHV recombinants was analyzed by restriction and Southern blot analyses, and the modified genome regions were further characterized by PCR and sequencing. These studies confirmed the desired mutations.

### Expression analysis of KHV-T ΔORF150 and KHV-T ΔORF150nat

To analyse the cellular response on KHV-T and the two *ORF150* deletion variants, CCB cells were infected with the respective viruses. Therefore, 6-well plates were seeded with 0.24 × 10^6^ cells per well. After reaching confluency, cells were infected with the respective virus at a MOI of 1. For downstream analysis samples were taken after 0, 6, 24, and 48 h post infection. All time points were prepared as triplicates.

### RNA extraction

Preparation of total RNA was performed with Trizol reagent (Invitrogen) and RNeasy mini spin columns (Qiagen). Cell pellets from 6-well plates were taken up in 500 µL Trizol and processed according to manufactures instructions. After phase separation, aqueous phase was mixed with ethanol (final concentration 50 %) and loaded onto mini spin columns of Qiagen RNeasy kit, and further processed according to the manufacturer’s instructions including DNase I treatment. Finally, RNA was quantified using NanoDrop ND-1000 machine (peqlab).

### Expression analysis by RT-qPCR

For reverse transcription QuantiTect Reverse Transcription kit (Qiagen) was used according to manufacturer’s protocol. In short, 1 µg of total RNA was used for gDNA wipe-out and reverse transcription using the supplied primer mix. The resulting 20 µL of cDNA were diluted with RNase-free water to 100 µL. In the following qPCR 2.5 µL of the diluted cDNA were mixed with 6.25 µL of 2x QuantiTect SYBR Green PCR Master Mix (Qiagen), 2.5 µL RNase-free water and 1.25 µL of the respective primer mix (3 µM). Cycling conditions were as following: 15 min of initial denaturation at 95 °C; denaturation: 15 s, 94 °C; annealing: 30 s, 60 °C; elongation: 30 s, 72 °C, fluorescence read out; with 40 cycles. Finally, a melt curve was recorded from 65 °C to 95 °C.

### Statistical analysis

Using Prism (GraphPad) mortality data were analysed and expressed as relative percentage survival^63^.

Gene expression was normalized with ddCt method^64^ using transcripts of 40 S ribosomal protein S11 and β-actin as standards. Statistical analysis was performed in Prism (GraphPad). Normalized expression levels were log2(*x*) transformed and tested for significance (*p* ≤ 0.05) by students t-test.

### Reporting summary

Further information on research design is available in the Nature Research Reporting Summary linked to this article.

## Supplementary information

REPORTING SUMMARY

## Data Availability

The data that support the findings of this study are available from the corresponding author upon reasonable request.
